# Conformal prediction for uncertainty quantification in dynamic biological systems

**DOI:** 10.1371/journal.pcbi.1013098

**Published:** 2025-05-12

**Authors:** Alberto Portela, Julio R. Banga, Marcos Matabuena

**Affiliations:** 1 Computational Biology Lab, MBG-CSIC (Spanish National Research Council), Pontevedra, Galicia, Spain; 2 Department of Biostatistics, Harvard University, Boston, Massachusetts, United States of America; Pázmány Péter Catholic University: Pazmany Peter Katolikus Egyetem, HUNGARY

## Abstract

Uncertainty quantification (UQ) is the process of systematically determining and characterizing the degree of confidence in computational model predictions. In systems biology, and particularly with dynamic models, UQ is critical due to the nonlinearities and parameter sensitivities that influence the behavior of complex biological systems. Addressing these issues through robust UQ enables a deeper understanding of system dynamics and more reliable extrapolation beyond observed conditions. Many state-of-the-art UQ approaches in this field are grounded in Bayesian statistical methods. While these frameworks naturally incorporate uncertainty quantification, they often require the specification of parameter distributions as priors and may impose parametric assumptions that do not always reflect biological reality. Additionally, Bayesian methods can be computationally expensive, posing significant challenges when dealing with large-scale models and seeking rapid, reliable uncertainty calibration. As an alternative, we propose using conformal predictions methods and introduce two novel algorithms designed for dynamic biological systems. These approaches can provide non-asymptotic guarantees, improving robustness and scalability across various applications, even when the predictive models are misspecified. Through several illustrative scenarios, we demonstrate that these conformal algorithms can serve as powerful complements—or even alternatives—to conventional Bayesian methods, delivering effective uncertainty quantification for predictive tasks in systems biology.

## Author summary

Uncertainty quantification involves determining how confident we are in the predictions made by mathematical models. This process is vital in the field of systems biology because it helps us understand and predict how these systems behave, despite their complexity. Typically, Bayesian statistics are used for this task. Although powerful, these methods often require specific prior information and make assumptions that may not always hold true for biological systems. Additionally, they struggle when we have limited data, and can be slow for large models. To address these issues, here we have developed two new algorithms based on conformal inference methods. These algorithms offer excellent reliability and scalability. Testing in various scenarios has demonstrated that they outperform traditional Bayesian methods, particularly when applied to large models. Our approach provides a new, general, and flexible method for quantifying uncertainty in dynamic biological models.

## Introduction

In the field of systems biology, we utilize mechanistic dynamic models as tools to analyze, predict and understand the intricate behaviors of complex biological processes [1–3]. Computational models are also increasingly being used to optimise treatment schedules and predict treatment responses in biomedicine (e.g. cancer therapy [4]), offering the potential to improve patient outcomes and personalise treatment strategies. These models are typically composed of sets of deterministic nonlinear ordinary differential equations, and are designed to provide a quantitative understanding of the dynamics which would be difficult to achieve through other means. In particular, this mechanistic approach offers several advantages over data-driven approaches [5,6]. First, it can generate more accurate predictions and can be applied to a broader range of situations. Second, it provides a deeper understanding of how the system works thanks to its mechanism-based nature, making it easier to interpret the reasons behind its behavior. Finally, it requires less data for training because it is based on established theories and principles that describe the underlying processes. Overall, mechanistic models can help in understanding the dynamics of biological systems, in predicting their behaviour under different conditions, in generating testable hypotheses, and in identifying knowledge gaps.

However, these benefits come with a trade-off. As the number of elements (species) and unknown variables in the system increases, the model becomes significantly more complex in terms of number of parameters and non-linear relationships. This complexity can make it difficult to interpret the model’s results [7], and damages its identifiability, i.e. the ability to uniquely determine the unknown parameters in a model from the available data. As the complexity increases with more species and unknown parameters, achieving full identifiability and observability becomes more difficult [8,9]. As a result, developing a reliable dynamic mechanistic model can be a demanding and error prone task, requiring considerable expertise and the use of comprehensive and systematic protocols [10–13]. Additionally, highly detailed models can lead to more uncertain predictions [14,15]. Model predictions are influenced by the uncertainty in parameters and their identifiability. Ideally, we should be able to characterise this impact in an interpretable manner, aiming to make useful predictions even when identifiability is poor [13,16]. Therefore, quantifying this uncertainty and how it affects different system states, a process known as Uncertainty Quantification (UQ) [17], is an open and fundamental challenge [18–21].

By doing so, UQ plays a key role in enhancing the reliability and interpretability of mechanistic dynamic models [22–25]. It helps in understanding the underlying uncertainties in the model parameters and predictions, thereby improving the model’s predictive power and its utility in decision-making processes [26,27]. Without proper UQ, models may become overconfident in their predictions, potentially leading to misleading results.

Different approaches for uncertainty quantification and robustness analysis in the context of systems biology have been reviewed elsewhere [22,28–30]. Roughly speaking, we can distinguish between Bayesian and frequentist methods [10–12,31–33]. Lately, the most predominant approach in the literature is to use Bayesian methods, which treat model parameters as random variables. Bayesian methods can perform well even with small sample sizes, especially when informative priors are used. Frequentist methods often require larger sample sizes to achieve reliable estimates. In practice, Bayesian approaches require parametric assumptions to define likelihood equations and the specification of a prior to derive an approximate posterior distribution of parameters for approximate and analytical methods [12,32]. This process is crucial for model estimation and inference.

However, Bayesian approaches often demand significant computational resources. Moreover, in the particular case of systems of differential equations, it is also typical to encounter identifiability issues, which result in multimodal posterior distributions that are challenging to handle in practice. In fact, although non-identifiabilities poses challenges to both frequentist and Bayesian sampling approaches; the latter can be especially susceptible to convergence failures [31,34–36]. Prediction profile likelihood methods and variants [13,33,37–39] provide a competitive alternative by combining a frequentist perspective with a maximum projection of the likelihood by solving a sequence of optimization problems. However, they can be computationally demanding when a large number of predictions must be assessed.

Although various uncertainty quantification approaches are utilized in the domain of systems biology, comparative assessments of the strengths and weaknesses of state-of-the-art methods remain scarce. Villaverde *et al* [21] recently presented a systematic comparison of four methods: Fisher information matrix (FIM), Bayesian sampling, prediction profile likelihood and ensemble modelling. The comparison was made considering case studies of increasing computational complexity. This assessment revealed an interplay between their applicability and statistical interpretability. The FIM method was not reliable, and the prediction profile likelihood did not scale up well, being very computationally demanding when a large number of predictions had to be assessed. An interesting trade-off between computational scalability and accuracy and statistical guarantees was found for the ensemble and Bayesian sampling approaches. The Bayesian method proved adequate for less complex scenarios; however, it faced scalability challenges and encountered convergence difficulties when applied to the more intricate problems. The ensemble approach presented better performance for large-scale models, but weaker theoretical justification.

Therefore, there is a clear need for UQ methods that possess both good scalability and strong theoretical statistical properties. Recently, in the literature of statistics and machine learning research, the use of conformal prediction [40] to quantify the uncertainty of model outputs has become increasingly popular as an alternative to Bayesian methods and other asymptotic approximations [41]. One of the successful aspects of this methodology in practice is the non-asymptotic guarantees that ensure the coverage of prediction regions is well-calibrated, at least from a global (marginal) perspective, as reviewed by [42]. However, to the best of our knowledge, their use in the systems biology and dynamical systems literature is not widespread, despite their expected promising properties for making predictions in complex biological systems.

Considering systems biology applications, we must account for the typically limited number of observations. In order to increase the statistical efficiency, we focus on conformal predictions based on the estimation of the conditional mean regression function [43], and the semi-parametric distributional location-scale regression models [44]. In order to exploit the specific structure of location-scale regression models, we propose two algorithms based on the jackknife methodology (see for example [45]). Recently, conformal prediction has also been extended to accommodate general statistical objects, such as graphs and functions that evolve over time, which can be very relevant in many biological problems [41,46].

The main contributions of our work are:

- We introduce and analyze two conformal prediction algorithms for dynamical systems, specifically tailored to optimize statistical efficiency under homoscedastic measurement errors or data transformations that approximate this condition:
  1. The first algorithm attains a target calibration quantile independently in each dimension of the system, providing flexibility in scenarios where the homoscedasticity assumption is not uniformly met.
  2. The second algorithm is designed for large-scale dynamical models. It globally standardizes the residuals and uses a single global quantile of calibration to construct the prediction regions, improving computational tractability and consistency across all dimensions.
- We empirically evaluate the proposed algorithms against traditional methods across multiple case studies of increasing complexity. The results highlight their favorable trade-offs in terms of statistical efficiency, computational runtime, and robustness, demonstrating their potential as effective alternatives to existing uncertainty quantification approaches in systems biology.

## 1. Methodology

### Modeling framework and notation

We consider dynamic models described by deterministic nonlinear ordinary differential equations (ODEs):

$$\dot{x}(t)=f(x(t),\theta ,t),\;x(t_{0})=x_{0},\;y(t)=g(x(t),\theta ,t),$$ (1)

where $x(t)\in {\mathbb{R}}^{n_{x}}$ is the vector of state variables at time *t*, $y(t)\in {\mathbb{R}}^{n_{y}}$ is the vector of observables, and $\theta \in {\mathbb{R}}^{n_{\theta }}$ is the vector of unknown parameters. The vector field $f:{\mathbb{R}}^{n_{x}}\times {\mathbb{R}}^{n_{\theta }}\times \mathbb{R}\mathrm{\backslash break}\to {\mathbb{R}}^{n_{x}}$ and the mapping $g:{\mathbb{R}}^{n_{x}}\times {\mathbb{R}}^{n_{\theta }}\times \mathbb{R}\to {\mathbb{R}}^{n_{y}}$ are possibly nonlinear functions.

To estimate θ in practice under ideal conditions, we use *n* observations $y_{1},\ldots ,y_{n}$ from the true model *y*(*t*) at times $t_{1},t_{2},\ldots ,t_{n}\subset ℐ=[0,T]$. The total number of measurements is $n\;\times \;n_{y}$. Due to measurement errors, in practice we do not observe $y_{1},\ldots ,y_{n}$ directly. Instead, we observe perturbed noisy observations from the deterministic process y(·):


$$\tilde{y}=(\begin{array}{cccc} {\tilde{y}}_{1,1} & {\tilde{y}}_{1,2} & \cdots & {\tilde{y}}_{1,n}\\ {\tilde{y}}_{2,1} & {\tilde{y}}_{2,2} & \cdots & {\tilde{y}}_{2,n}\\ \vdots & \vdots & \ddots & \vdots \\ {\tilde{y}}_{n_{y},1} & {\tilde{y}}_{n_{y},2} & \cdots & {\tilde{y}}_{n_{y},n} \end{array}).$$

We may apply a suitable data transformation to $\tilde{y}$ in order to achieve homoscedasticity in the transformed space. Specifically, each random observation is defined through the probabilistic model:

$$h_{k}({\tilde{y}}_{k,i},\lambda )=h_{k}(y_{k}(t_{i}),\lambda )+\epsilon_{k,i}=h_{k}(g_{k}(x(t_{i}),\theta ),\lambda )+\epsilon_{k,i},\;\left\{ \begin{array}{c} i=1,\ldots ,n,\\ k=1,\ldots ,n_{y},\\ \lambda \in {\mathbb{R}}^{n_{y}}. \end{array}\right.$$ (2)

where $\epsilon_{k,i}$ denotes zero-mean measurement noise, and $h_{k}(\cdot ,\lambda )$ is an increasing real-valued function depending on a shape parameter λ. A common example is the logarithmic function log(·), which corresponds to a log-normal probabilistic model [33]. For identifiability, we assume the variance of the observations with measurement error ${\tilde{y}}_{k,i}$ depends only on the regression function $g_{k}(x(t_{i}),\theta )$, thus encompassing specific heteroscedastic cases where the signal-to-noise ratio is homoscedastic in the transformed space but not in the original space.

Model (2) generalizes the Box-Cox transformation models for dynamical systems, known in the regression literature as the transform-both-sides (TBS) model [47].

For simplicity, we assume the measurement noise is independent across time points *t*_*i*_ and dimensions *k*, follows a normal distribution, and is homoscedastic across different dimensions:


$$\epsilon_{k,i}\sim 𝒩(0,\sigma_{k}^{2}),\;i=1,\ldots ,n;\;k=1,\ldots ,n_{y},$$

where $\sigma_{k}$ denotes the standard deviation for the *k*-th observable.

Throughout this manuscript, we present all modeling steps in a homoscedastic space directly in terms of the regression function *g* for clarity. Transformed versions of the algorithms involve applying the specified data transformation to the original sample $\tilde{y}$ and then running the algorithm in the transformed space, treating it as the homoscedastic case.

For simplicity in the explanations, we assume:


$$h_{k}(s,\lambda )=s,\;\forall s\in \mathbb{R},$$

which implies that *h*_*k*_ is the identity function. Therefore, we model the ODE systems directly from the original observations.

In our Gaussian noise setting, to achieve statistical efficiency in parameter estimation, we consider the maximum likelihood estimate (MLE) of the unknown parameters θ, denoted by $\hat{\theta }$. The MLE is obtained by minimizing the negative log-likelihood function:

$$\hat{\theta }=\arg\min_{\theta \in {\mathbb{R}}^{n_{\theta }}}ℒ(\theta ;{𝒟}_{n})=\frac{1}{2}\sum_{k=1}^{n_{y}}\sum_{i=1}^{n}\left[\log(2\pi \sigma_{k}^{2})+{\left(\frac{{\tilde{y}}_{k,i}-g_{k}(x(t_{i}),\theta )}{\sigma_{k}}\right)}^{2}\right],$$ (3)

which is a predominant optimization approach in the field of dynamical biological systems. Alternatively, when no information about the probabilistic mechanism of the random noise ϵ is available, one may minimize the mean squared error as an optimization criterion to estimate θ.

### 1.1. Conformal prediction for dynamical systems

In this section, we present new algorithms for conformal prediction tailored to the class of dynamical systems described earlier. The key idea is to treat the solution of the dynamical system as the regression function *g* (e.g., the conditional mean function), and to model the observed biological signals as being corrupted by measurement error ϵ. By utilizing the residuals, we can derive prediction regions using various conformal prediction strategies.

The main challenge in this type of regression is that the time series signals are observed at only a few (*n*) time points. Therefore, using full conformal methods—which require fitting n+1 models with n+1 observations each—is impractical due to the computational cost of optimizing the model parameters for large systems of differential equations. Alternatively, split conformal methods—which involve fitting models on random, disjoint subsets of data—are statistically inefficient when *n*<20, as is often the case in these biological problems.

To overcome the limitations of full and split conformal methods, we propose two new conformal prediction algorithms for dynamical systems. These methods enhance statistical efficiency in different scenarios by applying jackknife techniques [48].

Given a new i.i.d. observation $Y_{n+1}=g(x(t_{n+1}),\theta )+\epsilon_{n+1}$, and a confidence level α∈[0,1], our goal is to provide a prediction region $C^{\alpha }(t_{n+1})\subset {\mathbb{R}}^{n_{y}}$ such that


$$\mathbb{P}(Y_{n+1}\in C^{\alpha }(t_{n+1})|T=t_{n+1})=1-\alpha .$$

For practical purposes, we assume this prediction region exists and is unique.

Conformal prediction [40,42] is a general uncertainty quantification framework that provides non-asymptotic marginal (global) guarantees, independent of the underlying regression function *g*. Specifically, it ensures that


$$\mathbb{P}(Y_{n+1}\in {\hat{C}}^{\alpha }(t_{n+1}))\geq 1-\alpha ,$$

where the probability is over the random sample ${𝒟}_{n}\cup \{(t_{n+1},Y_{n+1})\}$.

There are three main variants of conformal prediction related to how the original sample ${𝒟}_{n}$ is partitioned [42,45]: full, split, and jackknife conformal methods.

To make a single prediction for a fixed pair $\left(t_{n+1},Y_{n+1}\right)$, full conformal methods use all n+1 observations and require fitting n+1 models—optimizing the parameters θ n+1 times—which is computationally intensive. Alternatively, split conformal methods are valid for any new data point $\left(t_{n+1},Y_{n+1}\right)$ and typically divide the sample into ⌊n/2⌋ observations to estimate *g* and the remaining n−⌊n/2⌋ observations to calibrate the prediction regions.

Finally, the jackknife approach serves as an intermediate method, making predictions for any data point $\left(t_{n+1},Y_{n+1}\right)$ without sacrificing statistical efficiency. This approach involves fitting *n* predictive models, each time excluding the *i*-th observation. To enhance the robustness of the conformal jackknife, we derive two algorithms based on [43] and the Jackknife+ method proposed in [45].

### 1.2. Algorithms

Algorithms 1 and 2 outline the core steps of our conformal uncertainty quantification (UQ) strategies for dynamical systems. In both algorithms, the first step involves excluding each *i*-th observation and fitting the regression functions to obtain jackknife residuals.

In the first algorithm, CUQDyn1, we apply a version of the conformal Jackknife+ method to each coordinate of the dynamical system, introducing flexibility when the uncertainty shape varies across dimensions. However, its theoretical convergence rates are often slower and require a larger number of observations compared to our second algorithm.

To address this and create a more efficient algorithm in certain homoscedastic cases, the second algorithm, CUQDyn2, assumes that the model is homoscedastic along each coordinate. In the second step, we standardize the residuals using an estimate of the standard deviation. We then consider the global quantile for calibration and, by re-scaling with the specific standard deviation, obtain the final prediction interval.

**Algorithm 1 Conformal naive UQ algorithm for dynamical systems (CUQDyn1).**

1. For each i=1,…,n, fit the model to the training data excluding the *i*-th point to obtain ${\hat{m}}_{-i}$. Compute the leave-one-out residual for each coordinate $k=1,\ldots ,n_{y}$ as $\epsilon_{i,k}=\left|y_{k}(t_{i})-{\hat{m}}_{-i,k}(t_{i})\right|$. Denote ${\hat{m}}_{.,k}(t_{i})$ as the vector containing the estimates for state *k* at time *t*_*i*_ from all ${\hat{m}}_{-i}$ fitted models, and denote $\epsilon_{.,k}$ as the vector of residuals for state *k* across all time points *t*_*i*_.
2. For each coordinate $k=1,\ldots ,n_{y}$ and i=1,…,n, output the prediction interval:
  $$\left(q_{\alpha }({\hat{m}}_{.,k}(t_{i})-\epsilon_{.,k}),\;q_{1-\alpha }({\hat{m}}_{.,k}(t_{i})+\epsilon_{.,k})\right),$$
  where α is the predictive level, and $q_{\alpha }$ and $q_{1-\alpha }$ denote the corresponding quantiles.

**Algorithm 2 Conformal global UQ algorithm for dynamical systems (CUQDyn2).**

1. For each i=1,…,n, fit the regression function ${\hat{m}}_{-i}$ to the training data with the *i*-th point removed, and compute the corresponding leave-one-out residual $\epsilon_{i,k}=\left|Y_{i}-{\hat{m}}_{-i,k}(t_{i})\right|$ for $k=1,\ldots ,n_{y}$.
2. For each coordinate $k=1,\ldots ,n_{y}$ and i=1,…,n, define the standardized variable $z_{i,k}=\frac{\epsilon_{i,k}}{\sigma_{k}}$, where $\sigma_{k}=\sqrt{\frac{1}{n}\sum_{j=1}^{n}\epsilon_{j,k}^{2}}$.
3. Calculate the quantile $q_{1-\alpha }$ for calibration for each coordinate $k=1,\ldots ,n_{y}$ using the sample $\{z_{i,k}{\}}_{k=1,i=1}^{n_{y},n}$.
4. Fit the regression function *m* to the full training data, and output the prediction interval for each coordinate:
  $$\mathrm{median}({\hat{m}}_{-1,k}(t_{i}),\ldots ,{\hat{m}}_{-n,k}(t_{i}))\pm q_{1-\alpha }\sigma_{k},\;k=1,\ldots ,n_{y}.$$

For the following theorem and all subsequent results, all probabilities are with respect to the distribution of the training data points $\left(t_{1},Y_{1}),\ldots ,(t_{n},Y_{n}\right)$ and the test data point $\left(t_{n+1},Y_{n+1}\right)$, drawn i.i.d. from an arbitrary distribution *P*. We implicitly assume that the regression method *m* is invariant to data ordering—that is, invariant to permutations. We treat the sample size n≥2 and the target coverage level α∈[0,1] as fixed throughout.

**Proposition 1:** The conformal jackknife prediction interval algorithms satisfy:


$$\mathbb{P}(Y_{n+1}\in {\hat{C}}^{\alpha }(t_{n+1}))\geq 1-2\alpha .$$

This follows from [45]. The target coverage level in the inequality is 1−α, which is often achieved except in certain non-trivial cases.

### 1.3. Computational complexity

In computational biology, large dynamical systems pose significant computational challenges. In our case, applying the conformal jackknife+ might involve a relatively large computational burden because the model must be run multiple times. For a dataset with *n* elements, we compute *n* quantities that depend on the complexity of estimating the regression function *m* over a grid of *n*_*i*_ observations along the *y*-coordinates.

When using a gradient-based approach for the Gaussian likelihood (in a non-optimized sense), the computational cost of gradient descent is typically O(k·n·p), where *k* is the number of iterations, *n* is the sample size, and *p* is the number of dimensions of the biological system.

Additionally, there is a cost associated with approximating the quantile function, which is typically O(nlogn). However, because the elements lie in a bounded range, this cost effectively reduces to *O*(*n*).

Hence, given that quantile estimation is linear in *n* for both algorithms, the overall computational complexity for the Gaussian likelihood with the jackknife+ strategy becomes:


$$[O(n)+O(knp)]\cdot O(n)\;=\;O(n^{2})+O(kn^{2}p),$$

which implies a quadratic cost in low-dimensional settings, dominated primarily by the sample size.

If both *n* and *p* grow large, the main computational bottleneck arises from estimating the regression function in leave-one-out validation. Recent computational strategies can help reduce the cost of repeated model estimations for each data point, see for example [49].

In the case of Stan with Hamiltonian Markov Chain Monte Carlo, the overall computational cost is O(S·k·n·p), where *S* denotes the number of posterior samples and *k* is the average number of leapfrog steps per iteration. In some scenarios, one might require up to 20,000 posterior samples, which can be more computationally demanding than running the algorithm n times in a frequentist setup to apply the jackknife. In many typical biological applications, the sample size n may be relatively small (e.g., n <100), and theoretical analysis suggests that under these conditions, conformal jackknife+ can often be considerably faster than a full Bayesian Markov Chain Monte Carlo procedure.

### 1.4. Advantages and limitations of the new conformal algorithms compared to existing Bayesian methods

In this study, we introduced two new conformal prediction algorithms, CUQDyn1 and CUQDyn2, for uncertainty quantification in nonlinear dynamical models of biological systems. These methods offer computational efficiency and practical advantages over existing Bayesian approaches, particularly in high-dimensional dynamical systems.

One of the primary advantages of our conformal methods is their computational efficiency. Compared to Bayesian methods—which often rely on computationally intensive Markov Chain Monte Carlo (MCMC) simulations—our algorithms are significantly faster, up to two orders of magnitude in our case studies (details in Sect 2). This efficiency makes them more practical for large-scale or high-dimensional models commonly encountered in systems biology.

Moreover, our conformal algorithms provide non-asymptotic coverage guarantees, ensuring that the prediction intervals maintain the desired coverage probability even with small sample sizes—a common scenario in biological experiments. They do not require tuning hyperparameters or specifying prior distributions, simplifying their application compared to Bayesian methods, which can be sensitive to prior choices and may require careful calibration.

However, there are limitations to our current approach that need to be acknowledged. First, our methods currently provide prediction intervals only for observed variables at observed time points. This means they are limited to predicting what has been measured and cannot directly predict unobserved states or future time points. In contrast, a primary aim in systems biology is often to predict unobserved states or dynamics beyond the available data. Extending our methods to handle such predictions is an area for future work.

Second, while it might appear that our methods require data with very good temporal resolution—necessitating dense observations for smooth interpolation—this is not necessarily the case. The conformal prediction framework does not inherently require densely sampled data. Our methods can be applied with sparse time points, although the precision of the prediction intervals may decrease with fewer observations. This depends on the estimation and validity of the parameters and the dynamical systems model. In practice, biological data often have limited time points, and our methods are designed to provide valid uncertainty quantification under such conditions. The use of jackknife techniques helps mitigate issues arising from small sample sizes, which is also a challenge in Bayesian methods and other statistical approaches for dynamical systems.

Third, concerns may arise regarding the width of the prediction intervals produced by our methods. It might seem that the intervals are too large—encompassing nearly all data points—which could be perceived as overly conservative. However, this can be a consequence of the finite-sample coverage guarantees provided by the conformal methods. The intervals are constructed to ensure the desired coverage probability (e.g., 95%), which may result in wider intervals, especially with small sample sizes or when the model is misspecified. Nevertheless, if the dynamical system is well-approximated, the lengths of the intervals are close to the optimal value, even with the non-asymptotic properties of our methods.

Regarding data requirements, our methods do not strictly require that all observables be measured at the same time points (i.e., matrix-like data). We only need to obtain a good approximation of the dynamical system’s solutions to achieve desirable properties. The conformal prediction framework can accommodate missing data and varying observation times for different variables. While synchronized measurements simplify implementation and interpretation, our methods are flexible and can adapt to the data structures commonly encountered in systems biology experiments.

In conclusion, our conformal prediction algorithms offer both computational efficiency and practical advantages for uncertainty quantification in systems biology, particularly for high-dimensional dynamical systems and scenarios where parameter distributions are not well-characterized. They provide non-asymptotic coverage guarantees without requiring hyperparameter tuning or prior distributions, ensuring robustness even when the underlying model estimations are not fully precise. Nevertheless, these methods have certain limitations, notably in predicting unobserved states or future time points and in capturing parameter uncertainty stemming from model fitting. Addressing these challenges will be a focus of future research.

It is important to recognize that no single method is universally optimal for all data analysis scenarios. However, our approaches can serve as powerful complements or alternatives to existing methods, offering valuable tools for a broad range of applications in systems biology and for example personalized medicine oncology applications.

### 1.5. Matlab implementation of the algorithms

We implemented our CUQDyn1 and CUQDyn2 algorithms in Matlab. Parameter estimations were formulated as the minimization of a least squares cost function, subject to the dynamics described by the model ODEs and parameter bounds. These non-convex problems were solved using a global hybrid method, enhanced scatter search (eSS), due to its good performance and robustness [50]. eSS is available in Matlab as part of the MEIGO optimization toolbox [51]. Our code also depends on the Optimization Toolbox and the Parallel Computing Toolbox. The software for the methodology and reproduction of the results is available at Zenodo (https://zenodo.org/doi/10.5281/zenodo.13644869). All computations were carried out on a Dell Precision 7920 workstation with dual Intel Xeon Silver 4210R processors.

### 1.6. Comparison with a Bayesian method

Bayesian methods are a classical approach for uncertainty quantification by estimating the posterior distribution $P(\theta |{𝒟}_{n})$, where θ represents the parameter of interest and ${𝒟}_{n}=\{X_{i}{\}}_{i=1}^{n}$ denotes the observed data. The key components in Bayesian analysis are the prior distribution P(θ), which encapsulates our initial beliefs about θ, and the likelihood function $P({𝒟}_{n}|\theta )$, which represents the probability of observing the data ${𝒟}_{n}$ given the parameter θ.

In many practical scenarios, computing the posterior distribution analytically is challenging. Markov Chain Monte Carlo (MCMC) methods provide general and powerful techniques to estimate the posterior distribution by generating samples from it. Notable MCMC algorithms include Metropolis-Hastings and Gibbs sampling.

Nowadays, general software tools are available for implementing Bayesian inference and MCMC methods, such as Stan. In Stan, models are defined in its modeling language by specifying the data, parameters, and the model (i.e., prior and likelihood). Stan can be seamlessly integrated with R through the rstan package [52], allowing users to perform Bayesian analyses within the R environment. The rstan package provides functions to compile Stan models, fit them to data, and extract samples for posterior analysis. Our implementations of the different case studies are also available at the Zenodo link above.

## 2. Results

The primary objective of this section is to assess the performance of our new conformal prediction methods against state-of-the-art approaches in well-established dynamical systems. By using simulation-based scenarios where the true system behavior is approximately known, we can evaluate how closely the computed prediction intervals reflect the intended coverage probabilities. Since we treat the dynamical system’s solution as the conditional mean regression function *g*, shorter prediction intervals naturally arise when the regression function is accurately estimated and the quantile calibration is properly tuned, ensuring that the resulting intervals are not overly conservative.

To achieve this, we consider four increasingly complex dynamic models (summarized in Table 1): (i) a simple logistic growth model, (ii) a two-species Lotka-Volterra predator-prey model, (iii) the well-known α-pinene kinetics model widely used in parameter estimation studies, and (iv) the challenging NF-κB signaling pathway. For each model, we generate synthetic datasets under various conditions—altering both data density and noise levels—to create parameter estimation problems that test the robustness of our methods. Except for the NF-κB case study, all states are fully observed.

**Table 1:** Summary of case study characteristics: number of unknown parameters ($n_{\theta }$), state variables (*n*_*x*_), and measured observables (*n*_*y*_).

	$n_{\theta }$	*n*_*x*_	*n*_*y*_
Logistic	2	1	1
Lotka-Volterra	4	2	2
α-Pinene	5	5	5
NFKB	29	15	6

The noisy observations are drawn according to the model defined in Equation 2:


$${\tilde{y}}_{k,i}=m_{k}(x(t_{i}),\theta )+\epsilon_{k,i},\;i=1,\ldots ,n;\;k=1,\ldots ,n_{y},$$

where $\epsilon_{k,i}$ are normally distributed errors:


$${\tilde{y}}_{k,i}\sim 𝒩(y_{k}(t_{i}),\sigma_{k}^{2}),\;\sigma_{k}=\epsilon \cdot \mu_{k},\;\mu_{k}=\frac{1}{n}\sum_{i=1}^{n}y_{k}(t_{i}).$$

Here, ϵ represents the percentage of added noise, and $\mu_{k}$ is the mean of the *k*-th state’s noise-free trajectory. Parameter estimation for the ODE models is performed via the maximum likelihood estimation (MLE) framework described by Equation (3), assuming Gaussian error distributions. We then compare our conformal methods against a Bayesian approach implemented in Stan to illustrate differences in performance and to highlight the advantages and potential limitations of our proposed uncertainty quantification strategies. All reported computation times were obtained on a PC with an Intel Xeon Silver 4210R processor running Windows 10 and Matlab R2023a.

### 2.1. Case I: Logistic growth model

As our initial case study we considered the well-known logistic model [53], governed by a single differential equation with two unknown parameters. This model is frequently used in population growth and epidemic spread modeling.

$$\dot{x}=rx\left(1-\frac{x}{K}\right).$$ (4)

Here, *r* represents the growth rate, and *K* denotes the carrying capacity. The initial condition considered in the generation of the datasets was x(0)=10. Additionally, the values of the parameters used were *r* = 0.1 and *K* = 100. Although the initial conditions are assumed to be known for all the case studies considered, they could be estimated without any significant challenges in obtaining the predictive regions using both algorithms proposed in this paper. Since this logistic model has an analytical solution, it facilitates the comparison of our methods’ performance with other established conformal methods for algebraic models, such as the jackknife+ [45].

To evaluate the performance of our methods on this case study, we considered various scenarios with different noise levels (0%, 1%, 5% and 10%) and dataset sizes (10, 20, 50 and 100 data points). For each combination of noise level and dataset size, we generated 50 different synthetic datasets, totaling 800 unique datasets. By generating multiple datasets for each scenario, we were able to obtain a robust estimate of the methods’ behavior and assess their consistency across different realizations of the data.

**Fig 1:**
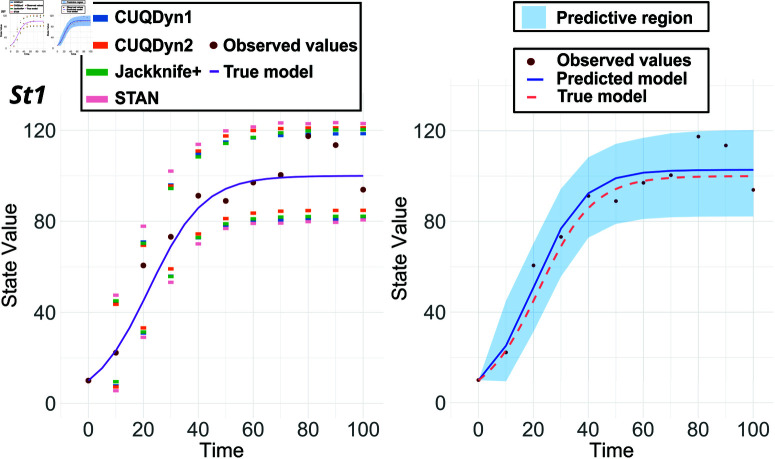
Comparative analysis of the Logistic model predictive regions: 95% predictive regions obtained from a 10-point dataset subjected to 10% noise. Left: results using four different methodologies: our two proposed methods (CUQDyn1 and CUQDyn2), the original jackknife+ method and a Bayesian approach, STAN. Right: predictive region obtained with the CUQDyn1 algorithm.

The comparative analysis of the logistic growth model, as shown in Fig 1, highlights the robustness of the proposed methods CUQDyn1 and CUQDyn2 compared to conventional methodologies such as the Bayesian approach implemented with STAN. For a 10-point synthetic dataset with a 10 percent noise level, the predictive regions obtained by both conformal methods showed good coverage without requiring prior calibration of the models, unlike the Bayesian approach. Moreover, both CUQDyn1 and CUQDyn2 yield predictive regions comparable to those generated by the jackknife+ method; however, in this particular case, the CUQDyn1 method shows superior performance.

In terms of computational efficiency, the conformal methods proved to be significantly faster than STAN, even for a problem of this small size. This makes them more suitable for real-time applications. A detailed comparison of computation times (which ranged from a few seconds to a few minutes) for datasets with different sizes and noise levels is provided in the Supporting Material.

**Fig 2:**
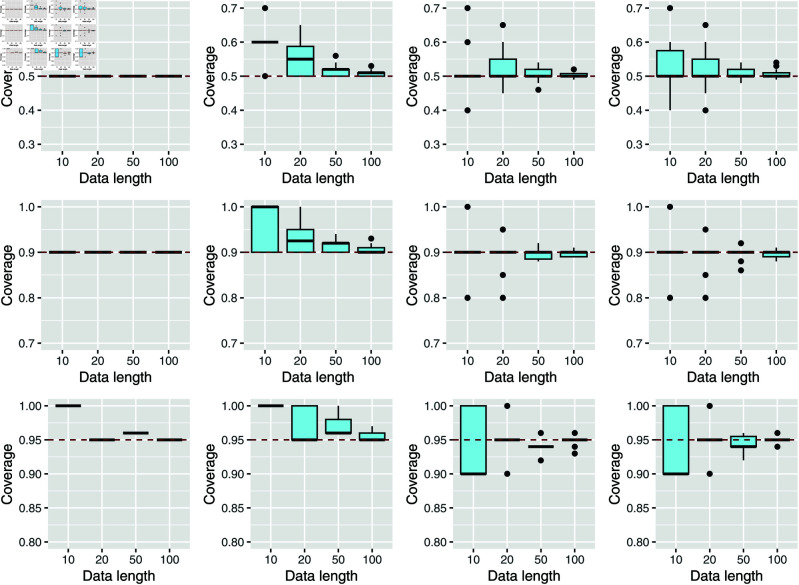
Boxplot of marginal coverage $\mathbb{P}(Y_{n+1}\in {\hat{C}}^{\alpha }(X_{n+1}))$ for different sample sizes and α=0.05, 0.1, and 0.5 of our first algorithm, CUQDyn1, for different noise levels (0%, 1%, 5%, and 10%). The results remain very stable across all examined cases.

In this example—exclusively here in the paper—we study the marginal coverage to examine the finite-sample properties $\mathbb{P}\left(Y_{n+1}\in {\hat{C}}^{\alpha }(X_{n+1})\right)$ for α = 0.05, 0.1, 0.5. We focus on the CUQDyn1 algorithm (see Fig 2) under various signal-to-noise conditions and sample sizes. The figure shows that our algorithm achieves good empirical performance, attaining the desired nominal level 1−α. In this setting, we do not need to consider the level 1−α/2, consistent with the guarantee provided by Proposition 1.

### 2.2. Case II: Lotka-Volterra model

As a second case study, we considered a two species Lotka-Volterra model [54], often referred to as the predator-prey model. This model provides a fundamental framework for studying the dynamics between two interacting species. In its simplest form, it describes the interactions between a predator species and a prey species through a set of coupled differential equations with four unknown parameters:

$${\dot{x}}_{1}=x_{1}(\alpha -\beta x_{2}),\;{\dot{x}}_{2}=-x_{2}(\gamma -\delta x_{1}).$$ (5)

Here, *x*_1_ and *x*_2_ represent the populations of the prey and predator, respectively. The parameters α, β, γ and δ are positive constants representing the interactions between the two species. Specifically, this parameters dictate the growth rates and interaction strengths, capturing the essence of biological interactions such as predation and competition. The initial conditions considered in the generation of the datasets were x(0)=(10,5). Additionally, the values of the parameters used were α=γ=0.5 and β=δ=0.02.

For this case study we generated datasets with the same noise levels (0%, 1%, 5% and 10%) as in the previous example and three different sizes (30, 60 and 120 points). Additionally, for each combination of noise level and dataset size, we generated 50 different synthetic datasets, resulting in a total of 600 unique datasets.

**Fig 3:**
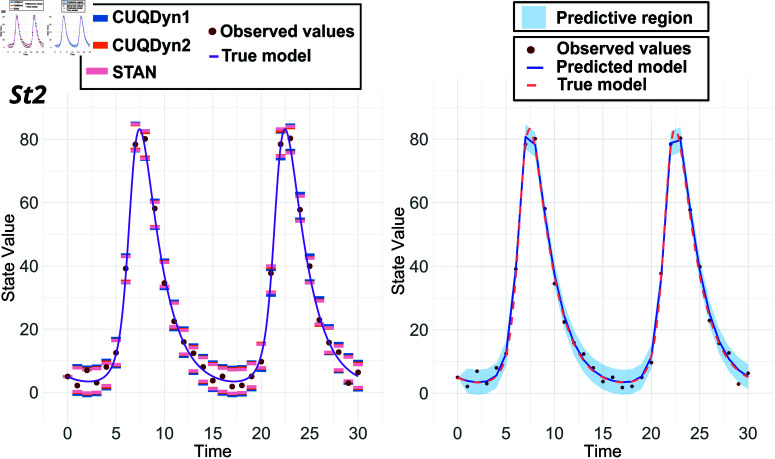
Comparative analysis of the Lotka-Volterra model: 95% predictive regions for *x*_2_ obtained from a 30-point dataset with 10% noise. Left: results using three different methodologies: our methods (CUQDyn1 and CUQDyn2) and STAN, a Bayesian approach. Right: predictive region obtained with CUQDyn2. Numerical details are available in Sect 1.2 of S1 Text.

Fig 3 shows the results in a 30-point Lotka-Volterra dataset, indicating that the predictive regions generated by the conformal methods and STAN are similar in terms of coverage. However, as in the previous case, CUQDyn1 and CUQDyn2 offer the advantage of not requiring extensive hyperparameter tuning, while also being more computationally efficient. In this particular example, while the Bayesian method obtains results within a timeframe on the order of minutes, both conformal methods achieve this in a significantly shorter span, on the order of seconds. A table comparing computation times across various datasets is provided in the Supporting Material.

### 2.3. Case III: Isomerization of α-Pinene

As a third case study, we examined the α-pinene isomerization model. The isomerization process of α-pinene is significant in industry, especially in the production of synthetic fragrances and flavors. These complex biochemical reactions can be effectively modeled using a system of five differential equations with five unknown parameters. The resulting kinetic model has been a classical example in the analysis of multiresponse data [55]. The kinetic equations encapsulate the transformation dynamics of α-pinene into its various isomers through a series of reaction steps:

$${\dot{x}}_{1}=-(p_{1}+p_{2})x_{1},\;{\dot{x}}_{2}=p_{1}x_{1},\;{\dot{x}}_{3}=p_{2}x_{1}-(p_{3}+p_{4})x_{3}+p_{5}x_{5},\;{\dot{x}}_{4}=p_{3}x_{3},\;{\dot{x}}_{5}=p_{4}x_{3}-p_{5}x_{5}.$$ (6)

In the equations above, each $p_{i}\in [0,1]$, i=1,…,5 represents a different rate of reaction, defining the conversion speed from one isomer to another. The initial conditions considered in the generation of the datasets were x(0)=(100,0,0,0,0). Additionally, the values of the parameters used were p=(5.93e−05,2.96e−05,2.05e−05,2.75e−04,4.00e−05). The dataset generation procedure for this case study mirrored that used for the Logistic model, employing the same noise levels and dataset sizes. Although we generated synthetic datasets to assess the method’s behavior, we illustrated this behavior with a real dataset from [55].

**Fig 4:**
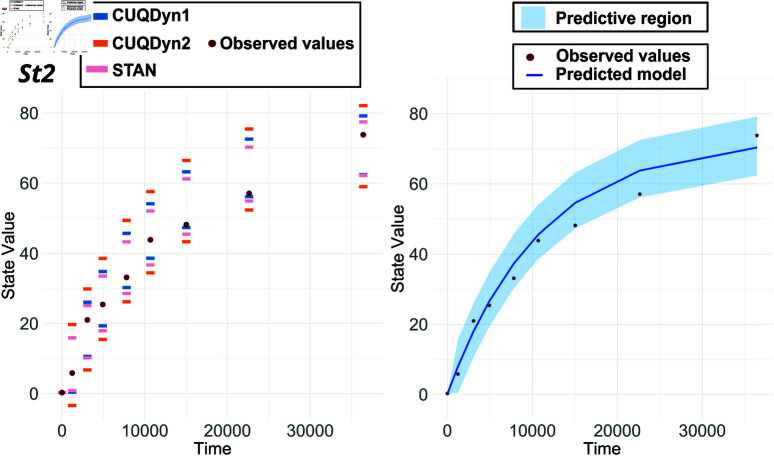
Comparative analysis of the α-pinene case: 95% predictive regions for *x*_2_ obtained from a 9-point real dataset. Left: predictive intervals using three different methodologies: (CUQDyn1, CUQDyn2) and STAN. Right: the predictive region using CUQDyn1. Numerical details are available in Sect 1.3 of S1 Text.

Fig 4 shows the resulting regions of the isomerization of α-Pinene by applying the different algorithms to the 9-point real dataset. The results are once again consistent between both conformal algorithms and closely align with the regions obtained using STAN. In terms of computational cost, the conformal algorithms are notably more efficient, requiring less than a minute to compute the regions, whereas the Bayesian approach takes several minutes. A comparison of execution times for the 9-point dataset is presented in Table 2. The results demonstrate that CUQDyn2 outperforms STAN considerably, completing computations in less than a minute compared to STAN’s several-minute runtime.

**Table 2:** Comparison of execution times (measured in seconds) for CUQDyn2 and STAN methods for a 9-point real dataset for the α-pinene isomerization model. The results for CUQDyn2 were obtained by averaging the execution times over 50 runs, while those for STAN were averaged over 5 runs. The table also includes the standard deviation of the execution times for each model.

	CUQDyn2	STAN
**Average Time**	5.180e+01	3.989e+02
**Standard Deviation**	2.880e+00	1.774e+01

### 2.4. Case IV: NFKB signaling pathway

The Nuclear Factor Kappa-light-chain-enhancer of activated B cells (NFKB) signaling pathway plays a key role in the regulation of immune response, inflammation and cell survival. This pathway is activated in response to various stimuli, including cytokines, stress and microbial infections, leading to the transcription of target genes involved in immune and inflammatory responses. Here we consider the dynamics of this pathway as described by a system of differential equations [56]:

$$\dot{IKKn}=kprod-kdeg\cdot IKKn\;-Tr\cdot k1\cdot IKKn,\;\dot{IKKa}=Tr\cdot k1\cdot IKKn-k3\cdot IKKa\;-Tr\cdot k2\cdot IKKa\cdot A20-kdeg\cdot IKKa\;-a2\cdot IKKa\cdot IkBa+t1\cdot IKKaIkBa\;-a3\cdot IKKa\cdot IkBaNFkB\;+t2\cdot IKKaIkBaNFkB,\;\dot{IKKi}=k3\cdot IKKa+Tr\cdot k2\cdot IKKa\cdot A20\;-kdeg\cdot IKKi,\;\dot{IKKaIkBa}=a2\cdot IKKa\cdot IkBa-t1\cdot IKKaIkBa,\;\dot{IKKaIkBaNFkB}=a3\cdot IKKa\cdot IkBaNFkB\;-t2\cdot IKKaIkBaNFkB,\;\dot{NFkB}=c6a\cdot IkBaNFkB-a1\cdot NFkB\cdot IkBa\;+t2\cdot IKKaIkBaNFkB-i1\cdot NFkB,\;\dot{NFkBn}=i1\cdot kv\cdot NFkB-a1\cdot IkBan\cdot NFkBn,\;\dot{A20}=c4\cdot A20t-c5\cdot A20,\;\dot{A20t}=c2+c1\cdot NFkBn-c3\cdot A20t,\;\dot{IkBa}=-a2\cdot IKKa\cdot IkBa\;-a1\cdot IkBa\cdot NFkB\;+c4a\cdot IkBat-c5a\cdot IkBa-i1a\cdot IkBa\;+e1a\cdot IkBan,\;\dot{IkBan}=-a1\cdot IkBan\cdot NFkBn+i1a\cdot kv\cdot IkBa\;-e1a\cdot kv\cdot IkBan,\;\dot{IkBat}=c2a+c1a\cdot NFkBn-c3a\cdot IkBat,\;\dot{IkBaNFkB}=a1\cdot IkBa\cdot NFkB-c6a\cdot IkBaNFkB\;-a3\cdot IKKa\cdot IkBaNFkB\;+e2a\cdot IkBanNFkBn,\;\dot{IkBanNFkBn}=a1\cdot IkBan\cdot NFkBn\;-e2a\cdot kv\cdot IkBanNFkBn,\;\dot{cgent}=c2c+c1c\cdot NFkBn-c3c\cdot cgent.$$ (7)

We assume that the available measurements are determined by the observation function $m:{\mathbb{R}}^{15}\to {\mathbb{R}}^{6}$, defined as:

m(·)=(NFkBn,IkBa+IkBaNFkB,A20t,IKKn+IKKa+IKKi,IKKa,IkBat). (8)

This means that, although the underlying system consists of 15 state variables and involves the estimation of 29 unknown parameters, only 6 of these states are directly observable. Such a significant discrepancy between the number of parameters and observables is a common challenge in systems biology and raises issues related to parameter identifiability. Identifiability refers to the ability to uniquely determine model parameters from the available data. When identifiability is limited, estimating unique parameter values may be infeasible; however, as discussed earlier, appropriate uncertainty quantification (UQ) methods can still provide valuable insights and support meaningful predictions despite these limitations.

The synthetic datasets in this scenario were generated using the same procedures applied in the previous case studies.

**Fig 5:**
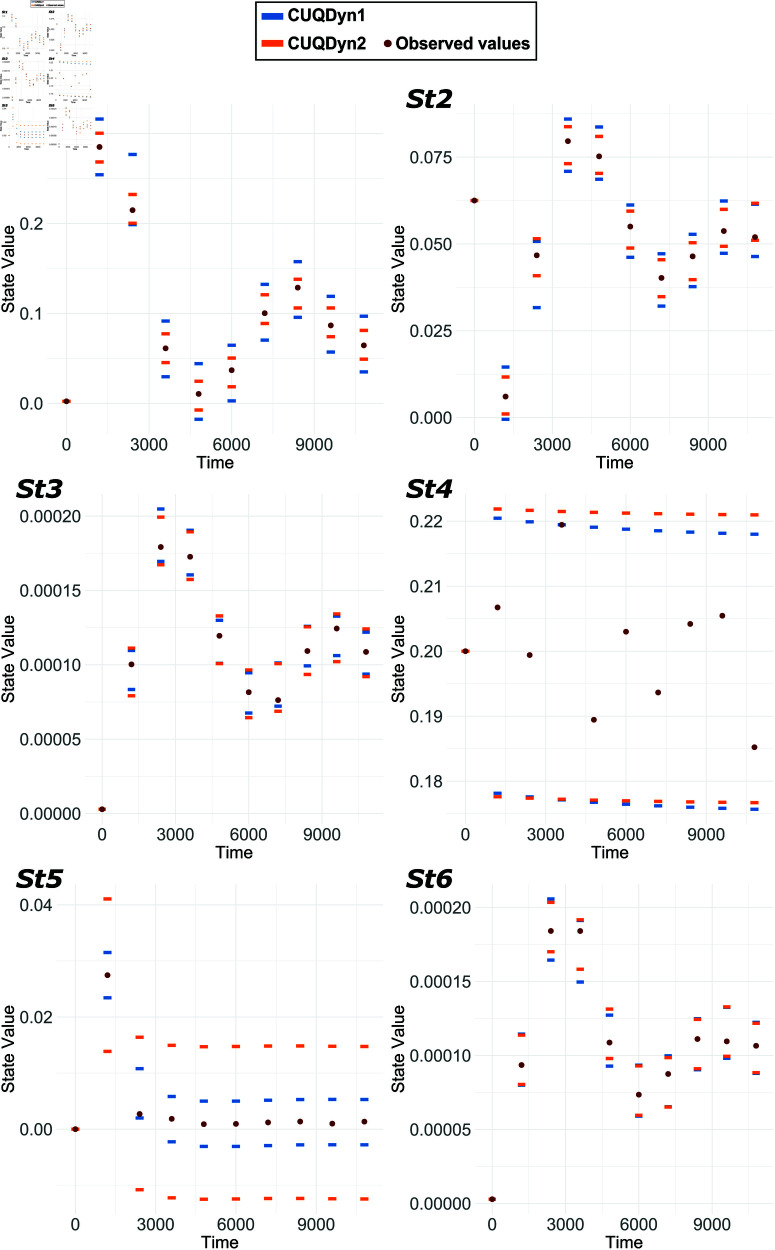
Comparative analysis of the NFkb signaling pathway model predictive regions. This figure presents the 95% predictive regions obtained from a 13-point synthetic dataset. It showcases the regions for the six observables obtained by using our two proposed methods: CUQDyn1 and CUQDyn2.

Fig 5 shows the results of applying our two methods to a 13-point synthetic dataset. Both methods based on conformal inference yielded results that are in close agreement with each other. However, in this case, we were not able to obtain adequate predictive regions using STAN, even after many hours of computation, probably due to the partial lack of identifiability. Remarkably, our CUQDyn1 and CUQDyn2 algorithms can compute the regions in just a few minutes using a standard PC. Detailed computation times are provided in the Supporting Material.

## 3. Discussion

In this study, we presented two algorithms, CUQDyn1 and CUQDyn2, based on conformal methods for uncertainty quantification in nonlinear dynamic models of biological systems. These methods enable the computation of prediction regions under the assumption that the signal-to-noise ratio is homoscedastic in the measurements or after applying a transformation to the original data. We compared the performance of these new methods with Bayesian approaches across a set of problems of increasing complexity. The main conclusions from our numerical results are summarized below.

Our algorithms were significantly faster—up to two orders of magnitude—than the Bayesian method implemented in Stan for the case studies examined. Without the need for hyperparameter tuning, our methods performed well and were in agreement with the Bayesian approach for smaller case studies and larger datasets (more than 50 time points). However, for high-dimensional biological systems, as illustrated with the NFKB case study, our conformal methods exhibited better accuracy, while Stan encountered convergence issues.

Moreover, our methods achieved good marginal coverage due to their non-asymptotic properties, even though they are not based on specified regression models. In contrast, Bayesian methods, exemplified by Stan, showed more significant impacts from poor calibration on marginal coverage, especially with small sample sizes and inadequate prior or likelihood specifications, due to the lack of non-asymptotic guarantees.

Our study also revealed that achieving good coverage properties with a Bayesian method requires careful tuning of the prior, which can be challenging even for well-known small problems and may be very difficult for new, larger problems in real applications. We encountered convergence issues with the MCMC strategy in Stan, likely due to the multimodal nature of posterior distributions and identifiability issues, consistent with previous reports [34].

A primary limitation of our new methods is that the prediction regions might occasionally include negative values when observed states are very close to zero, which may be unrealistic from a mechanistic perspective. This issue is observed in both the Stan Bayesian implementation and the conformal methods presented here. One potential cause is the assumption of homoscedasticity (i.e., consistent signal-to-noise ratio across the entire domain). Additionally, the underlying model may not be correctly specified across all parts of the domain.

A straightforward solution to this issue is to apply a prior data transformation, such as a log transformation, which allows for modeling heteroscedastic scenarios, or to use a more general family of possible transformations as introduced in model (2). To enhance the usability of our proposed methods within the scientific community, we have made the code and data from this study available in a public repository. Moving forward, we plan to offer updates, including optimized code for high-performance computing, novel validations, and automatic transformation approaches for various error structures in modeling.

Although our study primarily introduces new methods for uncertainty quantification in dynamic models, we also emphasize the importance of extracting biological insights from the results. The prediction regions generated by our methods can identify components of the biological systems (e.g. signalling pathway) with the most uncertainty, highlighting critical nodes or processes where variability may have significant biological implications. Specifically, analysis of these regions can:

- **Identify reliable predictions**: determine predictions with narrow regions (more reliable) versus those with high uncertainty. Low uncertainty predictions infer reliable mechanisms, while uncertain ones suggest areas for further experimental investigation.
- **Link predictions to experimental data**: cross-validate prediction regions with new experimental data to refine the model or propose new hypotheses.
- **Guide future experiments**: use uncertainty quantification to design experiments targeting uncertain or sensitive aspects, improving validation efficiency and optimizing data collection.

Overall, our study presents a new framework for uncertainty quantification in dynamical models using conformal prediction methods, which can be a promising alternative to classical Bayesian methods in systems biology. Notably, our new methods are computationally scalable, which is crucial for large biological models. From a statistical perspective, they offer non-asymptotic guarantees and avoid the technical difficulties of calibrating prior functions necessary in Bayesian statistics. For future work, we suggest exploring conformal quantile algorithms for large-scale dynamical biological systems [57]. These algorithms typically provide better conditional coverage than other conformal algorithms [58] and do not require the assumption of symmetric random errors. However, applying quantile conformal algorithms in practice may require collecting more temporal observations of dynamical systems, which might not always be feasible in real-world scenarios. Finally, in this work, we focus on examples involving different autonomous systems of differential equations, but the same methods can be applied to other types of dynamical systems, such as partial differential equations, or hybrid approaches that incorporate these approximations with deep learning, for example Neural ODEs [59].

## Supporting information

S1 Text Additional information supplementing this manuscript. (PDF)
