# Supplementary material for: Conformal prediction for uncertainty quantification in dynamic biological systems
Source: PLoS Comput Biol. 2025 May 12;21(5):e1013098. doi: 10.1371/journal.pcbi.1013098 (PMC12091895; doi:10.1371/journal.pcbi.1013098)
Supplement: S1 Text — (PDF) [file pcbi.1013098.s001.pdf]

SUPPORTING INFORMATION FOR "CONFORMAL PREDICTION FOR UNCERTAINTY QUANTIFICATION IN DYNAMIC BIOLOGICAL SYSTEMS"

Alberto Portela

Computational Biology Lab
MBG-CSIC, Spanish National Research Council
Pontevedra, Spain
alberto.portela@csic.es

Julio R. Banga

Computational Biology Lab
MBG-CSIC, Spanish National Research Council
Pontevedra, Spain
j.r.banga@csic.es

Marcos Matabuena

Department of Biostatistics
Harvard University
Cambridge, MA, USA
mmatabuena@hsph.harvard.edu

May 2, 2025

ABSTRACT

Here we provide supporting information, including technical details and additional results. The software for the methodology and the reproduction of the results is available at https://zenodo.org/doi/10.5281/zenodo.13644869.
Contact: mmatabuena@hsph.harvard.edu; j.r.banga@csic.es

1 Case studies: detailed results

All reported computation times were obtained on a PC with an Intel Xeon Silver 4210R processor running Windows 10 and Matlab R2023a.

1.1 Case I: Logistic growth model

Table A presents comprehensive numerical data from our comparative analysis of predictive regions for the Logistic model. These results corroborate our main findings, demonstrating that the conformal methods generate reliable predictive regions with performance comparable to established techniques. The data in Table 1 provides a quantitative

basis for assessing the effectiveness of various uncertainty quantification methods applied to the Logistic model. By examining metrics such as coverage probability and region size, we can observe how the conformal prediction algorithms perform relative to traditional approaches. This detailed breakdown allows for a more nuanced understanding of each method's strengths and limitations in the context of the Logistic model, supporting the broader conclusions drawn in the main text. Table B compares execution times for the logistic model on various datasets, showing that CUQDyn1 outperforms STAN even on this small-scale problem..

1.2 Case II: Lotka-Volterra model

Tables C and D provide numerical data from our comparative analysis of predictive regions for both states in the Lotka-Volterra model. The results demonstrate that the conformal algorithms generate predictive regions comparable to the Bayesian approach, while offering superior computational efficiency.A comparison of execution times for the Lotka-Volterra model across different datasets is presented in Table E. The results demonstrate that the CUQDyn2 method is considerably faster than STAN, completing computations in seconds compared to STAN's minutes-long timeframe.

1.3 Case III: Isomerization of α-Pinene

Figure A shows the resulting regions of the isomerization of α-Pinene by applying the different algorithms to the 9-point real dataset. The results are once again consistent between both conformal algorithms and closely align with the regions obtained using STAN. In terms of computational cost, the conformal algorithms are notably more efficient, requiring less than a minute to compute the regions, whereas the Bayesian approach takes several minutes.

In Tables F, G, H, I and J we present the numerical results corresponding to the comparative analysis of the α-Pinene isomerization model predictive regions for each of the five states. As observed in Figure A, the results demonstrate consistent predictive performance across all five states. This further underscores the reliability and agreement between the conformal algorithms and the Bayesian approach in capturing the predictive regions of the α-Pinene isomerization model.

Table A: Numerical results corresponding to the comparative analysis of the Logistic model predictive regions. This table shows the 95% lower and upper predictive bounds (LPB and UPB, respectively) obtained for each time point (t) in a 10-point dataset subjected to 10% noise. The observed data (y) and the true state value (x_{nom}) are also shown. The results are reported for four methodologies: the proposed `CUQDyn1` and `CUQDyn2` methods, the original jackknife+ approach, and a Bayesian method implemented with STAN.

			CUQDyn1		CUQDyn2		Jackknife+		STAN	
t	y	x_{nom}	LPB	UPB	LPB	UPB	LPB	UPB	LPB	UPB
0	1.000e+01	1.000e+01	1.000e+01	1.000e+01	1.000e+01	1.000e+01	1.000e+01	1.000e+01	1.000e+01	1.000e+01
10	2.226e+01	2.320e+01	7.698e+00	4.440e+01	7.213e+00	4.357e+01	9.493e+00	4.507e+01	5.545e+00	4.756e+01
20	6.066e+01	4.509e+01	3.092e+01	7.100e+01	3.318e+01	6.954e+01	3.140e+01	7.043e+01	2.904e+01	7.781e+01
30	7.327e+01	6.906e+01	5.593e+01	9.594e+01	5.916e+01	9.552e+01	5.578e+01	9.442e+01	5.324e+01	1.021e+02
40	9.123e+01	8.585e+01	7.284e+01	1.095e+02	7.453e+01	1.109e+02	7.296e+01	1.083e+02	7.015e+01	1.138e+02
50	8.895e+01	9.428e+01	7.816e+01	1.149e+02	8.116e+01	1.175e+02	7.888e+01	1.143e+02	7.681e+01	1.198e+02
60	9.703e+01	9.782e+01	7.997e+01	1.167e+02	8.357e+01	1.199e+02	8.105e+01	1.169e+02	7.905e+01	1.215e+02
70	1.004e+02	9.919e+01	8.055e+01	1.177e+02	8.441e+01	1.208e+02	8.181e+01	1.189e+02	7.917e+01	1.232e+02
80	1.174e+02	9.970e+01	8.073e+01	1.183e+02	8.468e+01	1.210e+02	8.207e+01	1.198e+02	7.982e+01	1.229e+02
90	1.135e+02	9.989e+01	8.079e+01	1.185e+02	8.477e+01	1.211e+02	8.215e+01	1.201e+02	7.953e+01	1.234e+02
100	9.390e+01	9.996e+01	8.081e+01	1.185e+02	8.480e+01	1.212e+02	8.218e+01	1.203e+02	8.060e+01	1.230e+02

Table B: Comparison of execution times (measured in seconds) for `CUQDyn1` and STAN methods for for datasets of varying sizes (10, 20, 50, and 100) and different noise levels (0%, 1%, 5%, and 10%) for the logistic model. The results for `CUQDyn1` were obtained by averaging the execution times over 50 runs, while those for STAN were averaged over 5 runs.

	CUQDyn1				STAN			
	10	20	50	100	10	20	50	100
0%	1.232e+01	1.867+01	4.286+01	2.057+02	1.658+01	2.312+01	5.961+01	2.624+02
1%	1.284e+01	1.889+01	4.412+01	2.062+02	1.542+01	2.256+01	5.972+01	2.650+02
5%	1.316e+01	1.902+01	4.365+01	2.063+02	1.536+01	2.374+01	6.180+01	2.649+02
10%	1.331e+01	1.900+01	4.397+01	2.082+02	1.681+01	2.417+01	6.237+01	2.657+02

Table C: Numerical results corresponding to the comparative analysis of the Lotka-Volterra model predictive regions for the first state. This table presents the 95% lower and upper predictive bounds (LPB and UPB, respectively) obtained for each time point (t) in a 30-point dataset subjected to 10% noise. The observed data (y) and the true state value (x_{nom}) are also shown. The results are reported for three methodologies: the proposed `CUQDyn1` and `CUQDyn2` methods and a Bayesian method implemented with STAN.

			CUQDyn1		CUQDyn2		STAN	
t	y	x_{nom}	LPB	UPB	LPB	UPB	LPB	UPB
0	1.000e+01	1.000e+01	1.000e+01	1.000e+01	1.000e+01	1.000e+01	1.000e+01	1.000e+01
1	1.627e+01	1.510e+01	9.974e+00	2.025e+01	9.535e+00	2.068e+01	1.022e+01	1.984e+01
2	2.597e+01	2.317e+01	1.804e+01	2.831e+01	1.760e+01	2.874e+01	1.832e+01	2.832e+01
3	3.373e+01	3.562e+01	3.046e+01	4.073e+01	3.000e+01	4.114e+01	3.069e+01	4.046e+01
4	5.374e+01	5.379e+01	4.854e+01	5.882e+01	4.807e+01	5.921e+01	4.837e+01	5.925e+01
5	7.607e+01	7.551e+01	7.009e+01	8.036e+01	6.961e+01	8.075e+01	6.965e+01	8.039e+01
6	7.493e+01	8.002e+01	7.344e+01	8.429e+01	7.304e+01	8.418e+01	7.317e+01	8.386e+01
7	4.005e+01	4.053e+01	3.381e+01	4.477e+01	3.347e+01	4.461e+01	3.364e+01	4.447e+01
8	1.435e+01	1.316e+01	7.461e+00	1.773e+01	7.070e+00	1.821e+01	7.415e+00	1.762e+01
9	6.658e+00	5.733e+00	2.295e-01	1.050e+01	-1.836e-01	1.096e+01	6.027e-01	1.044e+01
10	1.580e+00	3.786e+00	-1.595e+00	8.677e+00	-2.018e+00	9.122e+00	-1.310e+00	8.689e+00
11	6.848e+00	3.424e+00	-1.915e+00	8.357e+00	-2.340e+00	8.800e+00	-1.494e+00	8.226e+00
12	6.422e+00	3.821e+00	-1.528e+00	8.744e+00	-1.953e+00	9.187e+00	-1.257e+00	8.470e+00
13	4.250e+00	4.878e+00	-5.077e-01	9.764e+00	-9.314e-01	1.021e+01	-1.349e-01	9.607e+00
14	4.422e+00	6.777e+00	1.309e+00	1.158e+01	8.889e-01	1.203e+01	1.878e+00	1.148e+01
15	8.831e+00	9.921e+00	4.314e+00	1.459e+01	3.899e+00	1.504e+01	4.873e+00	1.474e+01
16	1.784e+01	1.498e+01	9.152e+00	1.942e+01	8.744e+00	1.988e+01	9.879e+00	1.965e+01
17	2.148e+01	2.297e+01	1.681e+01	2.708e+01	1.641e+01	2.755e+01	1.756e+01	2.741e+01
18	3.687e+01	3.532e+01	2.866e+01	3.893e+01	2.828e+01	3.942e+01	2.959e+01	3.937e+01
19	5.202e+01	5.337e+01	4.619e+01	5.667e+01	4.582e+01	5.696e+01	4.771e+01	5.748e+01
20	7.378e+01	7.512e+01	6.812e+01	7.858e+01	6.774e+01	7.888e+01	6.851e+01	7.887e+01
21	8.134e+01	8.039e+01	7.673e+01	8.701e+01	7.624e+01	8.738e+01	7.399e+01	8.524e+01
22	4.315e+01	4.139e+01	3.909e+01	4.999e+01	3.918e+01	5.032e+01	3.616e+01	4.739e+01
23	1.409e+01	1.343e+01	8.884e+00	1.916e+01	8.355e+00	1.950e+01	8.841e+00	1.841e+01
24	1.085e+01	5.804e+00	5.953e-01	1.087e+01	1.348e-01	1.128e+01	1.022e+00	1.065e+01
25	4.580e+00	3.804e+00	-1.537e+00	8.734e+00	-1.974e+00	9.166e+00	-1.097e+00	8.501e+00
26	-2.494e+00	3.423e+00	-1.973e+00	8.298e+00	-2.405e+00	8.736e+00	-1.860e+00	8.504e+00
27	1.040e+00	3.807e+00	-1.669e+00	8.602e+00	-2.098e+00	9.043e+00	-1.317e+00	8.542e+00
28	1.479e+00	4.849e+00	-7.498e-01	9.522e+00	-1.177e+00	9.964e+00	-2.282e-01	9.313e+00
29	1.030e+01	6.729e+00	9.260e-01	1.120e+01	5.132e-01	1.165e+01	1.641e+00	1.140e+01
30	9.117e+00	9.842e+00	3.710e+00	1.398e+01	3.310e+00	1.445e+01	4.792e+00	1.448e+01

Table D: Numerical results corresponding to the comparative analysis of the Lotka-Volterra model predictive regions for the second state. This table shows the 95% lower and upper predictive bounds (LPB and UPB, respectively) obtained for each time point (t) in a 30-point dataset subjected to 10% noise. The observed data (y) and the true state value (x_{nom}) are also shown. The results are reported for three methodologies: the proposed `CUQDyn1` and `CUQDyn2` methods and a Bayesian method implemented with STAN.

			CUQDyn1		CUQDyn2		STAN	
t	y	x_{nom}	LPB	UPB	LPB	UPB	LPB	UPB
0	5.000e+00	5.000e+00	5.000e+00	5.000e+00	5.000e+00	5.000e+00	5.000e+00	5.000e+00
1	-3.196e-02	3.883e+00	-1.135e+00	8.952e+00	-2.394e+00	1.019e+01	-1.270e+00	8.999e+00
2	5.589e+00	3.432e+00	-1.566e+00	8.521e+00	-2.830e+00	9.755e+00	-1.996e+00	8.629e+00
3	8.552e+00	3.716e+00	-1.242e+00	8.845e+00	-2.512e+00	1.007e+01	-1.394e+00	8.545e+00
4	4.930e+00	5.456e+00	5.887e-01	1.068e+01	-6.879e-01	1.190e+01	5.693e-02	1.060e+01
5	1.029e+01	1.208e+01	7.423e+00	1.751e+01	6.165e+00	1.875e+01	7.167e+00	1.778e+01
6	4.178e+01	3.702e+01	3.351e+01	4.366e+01	3.252e+01	4.510e+01	3.298e+01	4.470e+01
7	7.783e+01	7.820e+01	7.447e+01	8.551e+01	7.439e+01	8.697e+01	7.480e+01	8.636e+01
8	7.844e+01	7.709e+01	7.236e+01	8.332e+01	7.230e+01	8.488e+01	7.263e+01	8.453e+01
9	5.618e+01	5.556e+01	5.085e+01	6.130e+01	5.027e+01	6.286e+01	5.121e+01	6.225e+01
10	3.780e+01	3.691e+01	3.207e+01	4.217e+01	3.113e+01	4.371e+01	3.188e+01	4.271e+01
11	2.325e+01	2.402e+01	1.912e+01	2.921e+01	1.798e+01	3.056e+01	1.900e+01	2.969e+01
12	1.937e+01	1.565e+01	1.070e+01	2.079e+01	9.467e+00	2.205e+01	1.030e+01	2.094e+01
13	1.317e+01	1.034e+01	5.354e+00	1.544e+01	4.086e+00	1.667e+01	4.917e+00	1.561e+01
14	8.096e-01	7.036e+00	2.019e+00	1.211e+01	7.399e-01	1.333e+01	1.946e+00	1.230e+01
15	2.381e+00	5.030e+00	-1.277e-02	1.007e+01	-1.292e+00	1.129e+01	-2.094e-01	1.021e+01
16	1.747e+00	3.898e+00	-1.170e+00	8.917e+00	-2.445e+00	1.014e+01	-1.033e+00	9.236e+00
17	2.904e+00	3.435e+00	-1.674e+00	8.413e+00	-2.940e+00	9.645e+00	-1.793e+00	8.531e+00
18	1.409e+00	3.700e+00	-1.480e+00	8.607e+00	-2.732e+00	9.853e+00	-1.505e+00	9.062e+00
19	9.303e+00	5.394e+00	-9.628e-03	1.008e+01	-1.227e+00	1.136e+01	2.618e-01	1.056e+01
20	1.114e+01	1.184e+01	5.597e+00	1.568e+01	4.578e+00	1.716e+01	6.865e+00	1.730e+01
21	3.388e+01	3.622e+01	2.765e+01	3.862e+01	2.740e+01	3.998e+01	3.055e+01	4.150e+01
22	8.328e+01	7.771e+01	7.197e+01	8.306e+01	7.193e+01	8.452e+01	7.272e+01	8.509e+01
23	8.053e+01	7.745e+01	7.579e+01	8.654e+01	7.556e+01	8.814e+01	7.390e+01	8.533e+01
24	5.640e+01	5.599e+01	5.433e+01	6.441e+01	5.317e+01	6.576e+01	5.213e+01	6.339e+01
25	3.562e+01	3.722e+01	3.459e+01	4.468e+01	3.316e+01	4.575e+01	3.307e+01	4.380e+01
26	2.464e+01	2.423e+01	2.078e+01	3.087e+01	1.930e+01	3.188e+01	1.956e+01	3.038e+01
27	1.785e+01	1.578e+01	1.176e+01	2.185e+01	1.029e+01	2.288e+01	1.096e+01	2.153e+01
28	1.065e+01	1.042e+01	6.021e+00	1.611e+01	4.582e+00	1.717e+01	5.093e+00	1.595e+01
29	6.680e+00	7.087e+00	2.427e+00	1.251e+01	1.029e+00	1.361e+01	2.178e+00	1.252e+01
30	4.822e+00	5.060e+00	2.266e-01	1.031e+01	-1.136e+00	1.145e+01	7.373e-02	1.025e+01

Table E: Comparison of execution times (measured in seconds) for `CUQDyn2` and STAN methods for for datasets of varying sizes (30, 60 and 120) and different noise levels (0%, 1%, 5%, and 10%) for the Lotka-Volterra model. The results for `CUQDyn2` were obtained by averaging the execution times over 50 runs, while those for STAN were averaged over 5 runs.

	CUQDyn2			STAN		
	30	60	120	30	60	120
0%	3.061e+01	5.155e+01	3.186e+02	1.081e+02	2.646e+02	9.123e+02
1%	3.356e+01	5.216e+01	3.251e+02	1.142e+02	2.915e+02	9.081e+02
5%	3.328e+01	5.197e+01	3.249e+02	1.106e+02	2.877e+02	9.019e+02
10%	3.314e+01	5.520e+01	3.208e+02	1.218e+02	3.074e+02	9.003e+02

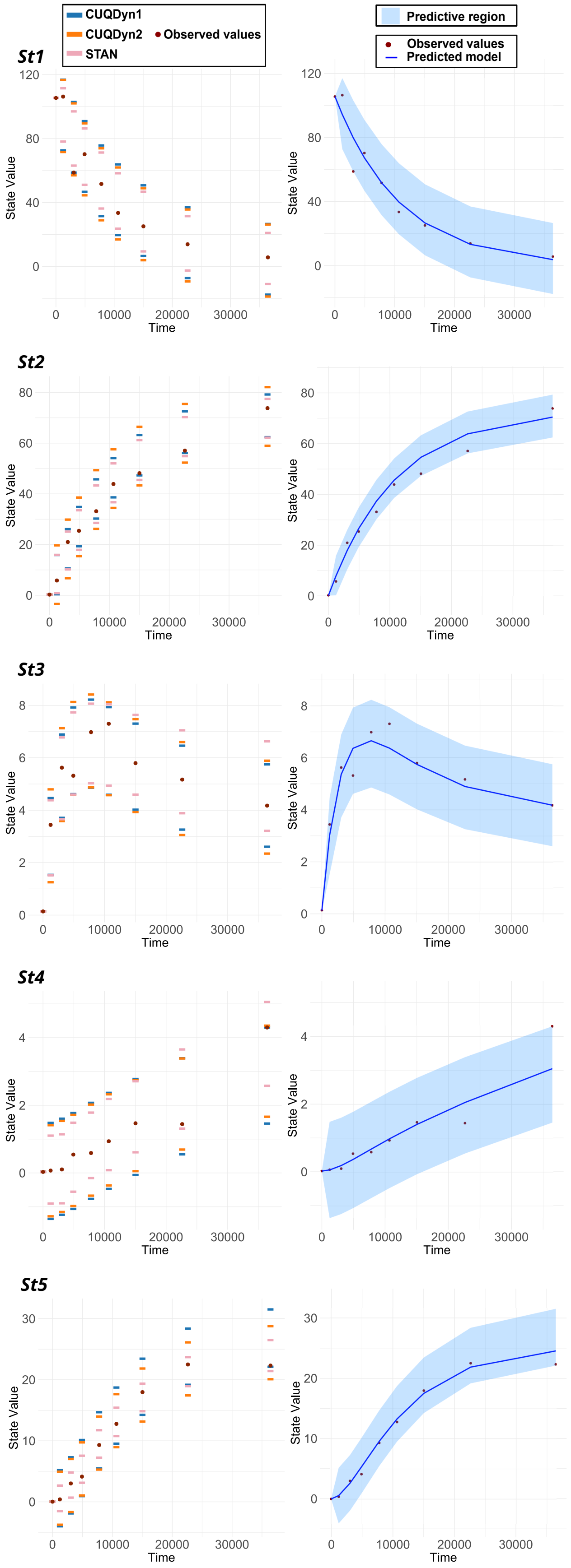

Figure A: Comparative analysis of the α-pinene isomerization model predictive regions. This figure shows the 95% predictive regions obtained from a 9-point real dataset. It showcases the regions for the first two states obtained by using three different methodologies: our two proposed methods (CUQDyn1 and CUQDyn2) and a Bayesian approach implemented with STAN.

Table F: Numerical results corresponding to the comparative analysis of the α-pinene isomerization model predictive regions for the first state. This table displays the observed data (y) alongside the 95% lower and upper predictive bounds (LPB and UPB, respectively) calculated for each time point (t) in a 9-point real dataset. The results are reported for three methodologies: the proposed `CUQDyn1` and `CUQDyn2` methods and a Bayesian method implemented with STAN.

		CUQDyn1		CUQDyn2		STAN	
t	y	LPB	UPB	LPB	UPB	LPB	UPB
0	1.054e+02	1.054e+02	1.054e+02	1.054e+02	1.054e+02	1.054e+02	1.054e+02
1230	1.063e+02	7.266e+01	1.169e+02	7.162e+01	1.167e+02	7.814e+01	1.115e+02
3060	5.875e+01	5.875e+01	1.030e+02	5.702e+01	1.020e+02	6.308e+01	9.701e+01
4920	7.023e+01	4.670e+01	9.091e+01	4.450e+01	8.953e+01	5.110e+01	8.633e+01
7800	5.161e+01	3.149e+01	7.570e+01	2.888e+01	7.391e+01	3.624e+01	7.128e+01
10680	3.350e+01	1.965e+01	6.385e+01	1.691e+01	6.194e+01	2.364e+01	5.830e+01
15030	2.510e+01	6.523e+00	5.073e+01	3.894e+00	4.892e+01	9.418e+00	4.676e+01
22620	1.388e+01	-7.284e+00	3.693e+01	-9.388e+00	3.564e+01	-2.531e+00	3.148e+01
36420	5.688e+00	-1.763e+01	2.658e+01	-1.883e+01	2.620e+01	-1.103e+01	2.096e+01

Table G: Numerical results corresponding to the comparative analysis of the α-pinene isomerization model predictive regions for the second state. This table displays the observed data (y) alongside the 95% lower and upper predictive bounds (LPB and UPB, respectively) calculated for each time point (t) in a 9-point real dataset. The results are reported for three methodologies: the proposed `CUQDyn1` and `CUQDyn2` methods and a Bayesian method implemented with STAN.

		CUQDyn1		CUQDyn2		STAN	
t	y	LPB	UPB	LPB	UPB	LPB	UPB
0	2.769e-01	2.769e-01	2.769e-01	2.769e-01	2.769e-01	2.769e-01	2.769e-01
1230	5.842e+00	4.078e-01	1.589e+01	-3.424e+00	1.970e+01	8.377e-01	1.593e+01
3060	2.101e+01	1.059e+01	2.608e+01	6.730e+00	2.985e+01	1.020e+01	2.511e+01
4920	2.542e+01	1.934e+01	3.482e+01	1.543e+01	3.855e+01	1.792e+01	3.353e+01
7800	3.314e+01	3.024e+01	4.573e+01	2.621e+01	4.933e+01	2.855e+01	4.330e+01
10680	4.389e+01	3.862e+01	5.410e+01	3.444e+01	5.756e+01	3.669e+01	5.201e+01
15030	4.814e+01	4.729e+01	6.321e+01	4.333e+01	6.645e+01	4.547e+01	6.119e+01
22620	5.705e+01	5.611e+01	7.253e+01	5.231e+01	7.543e+01	5.487e+01	7.025e+01
36420	7.378e+01	6.237e+01	7.918e+01	5.897e+01	8.209e+01	6.217e+01	7.745e+01

Table H: Numerical results corresponding to the comparative analysis of the α-pinene isomerization model predictive regions for the third state. This table displays the observed data (y) alongside the 95% lower and upper predictive bounds (LPB and UPB, respectively) calculated for each time point (t) in a 9-point real dataset. The results are reported for three methodologies: the proposed `CUQDyn1` and `CUQDyn2` methods and a Bayesian method implemented with STAN.

		CUQDyn1		CUQDyn2		STAN	
t	y	LPB	UPB	LPB	UPB	LPB	UPB
0	1.409e-01	1.409e-01	1.409e-01	1.409e-01	1.409e-01	1.409e-01	1.409e-01
1230	3.441e+00	1.535e+00	4.463e+00	1.253e+00	4.796e+00	1.515e+00	4.380e+00
3060	5.622e+00	3.709e+00	6.887e+00	3.584e+00	7.127e+00	3.656e+00	6.776e+00
4920	5.316e+00	4.615e+00	7.918e+00	4.583e+00	8.125e+00	4.601e+00	7.730e+00
7800	6.977e+00	4.858e+00	8.217e+00	4.869e+00	8.411e+00	5.025e+00	8.063e+00
10680	7.298e+00	4.590e+00	7.931e+00	4.574e+00	8.117e+00	4.936e+00	8.036e+00
15030	5.795e+00	4.021e+00	7.303e+00	3.928e+00	7.470e+00	4.595e+00	7.635e+00
22620	5.169e+00	3.261e+00	6.461e+00	3.055e+00	6.598e+00	3.885e+00	7.048e+00
36420	4.175e+00	2.608e+00	5.748e+00	2.346e+00	5.889e+00	3.215e+00	6.627e+00

Table I: Numerical results corresponding to the comparative analysis of the α-pinene isomerization model predictive regions for the fourth state. This table displays the observed data (y) alongside the 95% lower and upper predictive bounds (LPB and UPB, respectively) calculated for each time point (t) in a 9-point real dataset. The results are reported for three methodologies: the proposed `CUQDyn1` and `CUQDyn2` methods and a Bayesian method implemented with STAN.

		CUQDyn1		CUQDyn2		STAN	
t	y	LPB	UPB	LPB	UPB	LPB	UPB
0	3.034e-02	3.034e-02	3.034e-02	3.034e-02	3.034e-02	3.034e-02	3.034e-02
1230	6.992e-02	-1.359e+00	1.482e+00	-1.283e+00	1.411e+00	-9.063e-01	1.103e+00
3060	1.027e-01	-1.237e+00	1.604e+00	-1.156e+00	1.539e+00	-8.984e-01	1.143e+00
4920	5.430e-01	-1.065e+00	1.776e+00	-9.783e-01	1.716e+00	-5.551e-01	1.485e+00
7800	5.887e-01	-7.679e-01	2.073e+00	-6.727e-01	2.022e+00	-1.521e-01	1.784e+00
10680	9.356e-01	-4.725e-01	2.368e+00	-3.696e-01	2.325e+00	8.084e-02	2.190e+00
15030	1.468e+00	-6.209e-02	2.779e+00	5.318e-02	2.747e+00	6.100e-01	2.711e+00
22620	1.443e+00	5.493e-01	3.390e+00	6.911e-01	3.385e+00	1.312e+00	3.652e+00
36420	4.301e+00	1.460e+00	4.301e+00	1.662e+00	4.356e+00	2.578e+00	5.056e+00

Table J: Numerical results corresponding to the comparative analysis of the α-pinene isomerization model predictive regions for the fifth state. This table displays the observed data (y) alongside the 95% lower and upper predictive bounds (LPB and UPB, respectively) calculated for each time point (t) in a 9-point real dataset. The results are reported for three methodologies: the proposed `CUQDyn1` and `CUQDyn2` methods and a Bayesian method implemented with STAN.

		CUQDyn1		CUQDyn2		STAN	
t	y	LPB	UPB	LPB	UPB	LPB	UPB
0	3.252e-02	3.252e-02	3.252e-02	3.252e-02	3.252e-02	3.252e-02	3.252e-02
1230	4.046e-01	-4.002e+00	5.192e+00	-3.748e+00	4.940e+00	-1.514e+00	2.666e+00
3060	3.011e+00	-1.894e+00	7.300e+00	-1.668e+00	7.020e+00	6.917e-01	4.812e+00
4920	4.136e+00	9.374e-01	1.013e+01	1.051e+00	9.739e+00	3.127e+00	7.557e+00
7800	9.308e+00	5.485e+00	1.468e+01	5.285e+00	1.397e+01	7.238e+00	1.173e+01
10680	1.277e+01	9.528e+00	1.872e+01	8.952e+00	1.764e+01	1.078e+01	1.542e+01
15030	1.797e+01	1.426e+01	2.345e+01	1.315e+01	2.184e+01	1.484e+01	1.935e+01
22620	2.250e+01	1.917e+01	2.837e+01	1.743e+01	2.612e+01	1.895e+01	2.370e+01
36420	2.232e+01	2.212e+01	3.152e+01	2.008e+01	2.877e+01	2.142e+01	2.650e+01

Table K: Parameter estimation comparison for the first three case studies. Since the parameter estimation process is identical for both algorithms proposed in this paper, for simplicity, we will refer to the parameters obtained with them as CUQDyn. For the case study on the isomerization of α-pinene, a real dataset was considered, and thus the nominal parameters for this problem are unknown.

	Logistic		Lotka-Volterra				α-Pinene				
	r	K	α	β	γ	δ	p_1	p_2	p_3	p_4	p_5
True	1.000e-01	1.000e+02	5.000e-01	2.000e-02	5.000e-01	2.000e-02	NA	NA	NA	NA	NA
STAN	1.152e-01	1.021e+02	4.992e-01	2.004e-02	5.000e-01	2.001e-02	5.998e-05	2.797e-05	1.860e-05	2.826e-04	5.366e-05
CUQDyn	1.112e-01	1.027e+02	4.984e-01	2.001e-02	5.001e-01	2.000e-02	6.307e-05	2.841e-05	1.609e-05	2.729e-04	4.365e-05

1.4 Case IV: NFKB signaling pathway

The parameter values used in the generation of the datasets are as follows:

$$\begin{aligned}
\mathrm{a1} &= 5\times10^{-1}, & \mathrm{a2} &= 2\times10^{-1}, & \mathrm{t1} &= 1\times10^{-1},\\
\mathrm{a3} &= 1\times10^{0}, & \mathrm{t2} &= 1\times10^{-1}, & \mathrm{c1a} &= 5\times10^{-7},\\
\mathrm{c2a} &= 0\times10^{0}, & \mathrm{c3a} &= 4\times10^{-4}, & \mathrm{c4a} &= 5\times10^{-1},\\
\mathrm{c5a} &= 1\times10^{-4}, & \mathrm{c6a} &= 2\times10^{-5}, & \mathrm{c1} &= 5\times10^{-7},\\
\mathrm{c2} &= 0\times10^{0}, & \mathrm{c3} &= 4\times10^{-4}, & \mathrm{c4} &= 5\times10^{-1},\\
\mathrm{c5} &= 3\times10^{-4}, & \mathrm{k1} &= 2.5\times10^{-3}, & \mathrm{k2} &= 1\times10^{-1},\\
\mathrm{k3} &= 1.5\times10^{-3}, & \mathrm{kprod} &= 2.5\times10^{-5}, & \mathrm{kdeg} &= 1.25\times10^{-4},\\
\mathrm{kv} &= 5\times10^{0}, & \mathrm{i1} &= 2.5\times10^{-3}, & \mathrm{e2a} &= 1\times10^{-2},\\
\mathrm{i1a} &= 1\times10^{-3}, & \mathrm{e1a} &= 5\times10^{-4}, & \mathrm{c1c} &= 5\times10^{-7},\\
\mathrm{c2c} &= 0\times10^{0}, & \mathrm{c3c} &= 4\times10^{-4}.
\end{aligned}$$

A comparison of execution times for the NFKB signaling pathway model, using a 13-point synthetic dataset, is presented in Table L. The results demonstrate that CUQDyn1 is considerably faster than STAN. STAN was unable to produce results within a reasonable time, whereas CUQDyn1 completed the computations in minutes.

Table L: Comparison of execution times (measured in seconds) for `CUQDyn1` and STAN methods for a 13-point synthetic dataset for the NFKB signaling pathway model. The results for `CUQDyn1` were obtained by averaging the execution times over 50 runs. STAN did not converge to a solution even after 24 hours of computation.

	**CUQDyn1**	**STAN**
**Average Time (s)**	2.577e+02	———
**Standard Deviation**	1.114e+02	———
